# A paper-based polystyrene/nylon Janus platform for the microextraction of UV filters in water samples as proof-of-concept

**DOI:** 10.1007/s00604-021-05047-x

**Published:** 2021-10-25

**Authors:** Juan L. Benedé, Alberto Chisvert, Rafael Lucena, Soledad Cárdenas

**Affiliations:** 1 Affordable and Sustainable Sample Preparation (AS2P) Research Group, Departamento de Química Analítica, Instituto Universitario de Investigación en Química Fina y Nanoquímica IUNAN, Universidad de Córdoba, Campus de Rabanales, Edificio Marie Curie (anexo), E-14071 Córdoba, Spain; 2 GICAPC Research Group, Department of Analytical Chemistry, University of Valencia, 46100 Burjassot, Valencia Spain

**Keywords:** Environmental analysis, Janus platform, Mixed-mode chemistry platform, Cellulose paper-based microextraction, Sustainable synthesis, UV filters, LC-MS/MS

## Abstract

A new mix-mode cellulose-based sorptive phase is described that combines two different polymeric domains (i.e., nylon and polystyrene), thus providing simultaneous hydrophilic and hydrophobic features as a result. By analogy with Janus materials, the new paper-based sorptive phase has been named paper-based polystyrene/nylon Janus-platform (P-Ps/Ny-JP). The main advantages of the proposed P-Ps/Ny-JP are the sustainability, simplicity in synthesis, and low cost of this extraction device. The main parameters affecting the synthesis (i.e., coating procedure and polymers proportion) have been studied, and the resulting material has been characterized via scanning electron microscopy and infrared spectroscopy. As a proof-of-concept, the simultaneous extraction of fourteen UV filters of a wide range of polarity, with log *P* values ranging from − 0.234 to 16.129, from water samples and their determination by liquid chromatography-tandem mass spectrometry (LC–MS/MS) has been performed. The proposed methodology enables the determination of these chemicals with limits of detection from 12 to 71 ng L^−1^, and the precision, expressed as a relative standard deviation, was below 15%. The extraction device was applied to the analysis of real water samples likely to contain UV filters (i.e., two private swimming pool water and one seawater) and the recovery values were in the range 73–121%.

**Graphical abstract:**

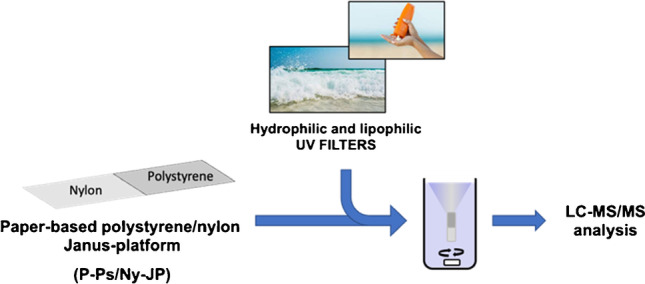

**Supplementary Information:**

The online version contains supplementary material available at 10.1007/s00604-021-05047-x.

## Introduction

The synthesis of new sorptive phases is one of the leading research lines in the field of microextraction techniques. Green analytical chemistry principles [1] suggest using renewable resources and natural materials in their design [2, 3]. Paper-based sorptive phases have demonstrated their potential, which relies on the sustainability and low price of the material and the versatility of the coatings. These phases were reported for the first time by Hurtubise and coworkers in the 1990s [4, 5], but their potential in microextraction has remained almost unexplored for two decades. The flat shape of the paper, formed by the irregular packing of the cellulose fibers, makes it attractive in the thin-film microextraction technique [6, 7]. Although cellulose structure may lead to consider paper as an inert material, studies from Meng et al. [8] and Diaz-Liñan et al. [9] have demonstrated the capacity of unmodified paper to extract analytes. The paper surface can be covalently modified [10–13] or physically coated with a sorptive phase [14–19] for better performance. In the latter approach, dip-coating and drop-casting have emerged as useful synthesis techniques. They have allowed the preparation of papers coated with polymers [14–16], nanomaterials [19], or their combination (nanocomposites) [17, 18]. Other approaches, like the partial carbonization of paper [20], have been recently proposed to boost the extraction capacity of raw paper.

A high selectivity is desirable when one compound or a family of structurally related compounds are targeted. However, an excessive selectivity may drive to chemical information losses when substances covering a wide range of chemical properties are intended to be determined in the same analysis. This situation is typical in non-targeted analysis (e.g., untargeted metabolomics) [21] or when a group of chemicals having a different structure but similar effect/use (e.g., endocrine disrupters, UV filters) is targeted. In such cases, the simultaneous and effective isolation of the analytes becomes challenging. In a previous article [22], we have demonstrated that the cellulose substrate and a polyamide coating can synergically contribute to the extraction capacity allowing the joint isolation of analytes with logarithms of octanol/water partition coefficient (log P_ow_) in the range from 0.88 to 2.83. However, for a wider polarity interval, a single sorbent phase would not be versatile enough.

Janus materials present an intentionally designed anisotropy that can affect their shape or chemical structure [23]. These materials, which can comprise particle and flat shapes, have found application in many fields [24, 25], including biomedicine [26]. In particular, Janus papers consist of a flat cellulose substrate where two or more chemically different domains are found [27, 28]. In the analytical chemistry realm, Janus papers are especially interesting in electrochemistry, where the simultaneous measurement of several analytes in a single and miniaturized platform can be conducted [29, 30]. However, the use of Janus papers in sample preparation has not been explored yet. To fill this gap, in the present article, a mix-mode paper-based sorptive phase is presented. It combines two different polymeric domains (i.e., nylon and polystyrene), providing simultaneous hydrophilic and hydrophobic features as a result. By analogy with Janus materials, the new paper-based sorptive phase has been named paper-based polystyrene/nylon Janus platform (P-Ps/Ny-JP). As a proof-of-concept, the trace determination of fourteen organic UV filters of a very wide polarity range (log *P*_ow_ values from − 0.234 to 16.129) in environmental water samples was considered for showing the potential and capability of this new extraction platform.

UV filters, which are added not only to cosmetics but also to other manufactured products to prevent or reduce the harmful effects of solar radiation [31], can reach the environment by direct and/or indirect sources and are being (bio)accumulated in aquatic ecosystems and living organisms [32]. Due to the harmful effects induced by these compounds on the aquatic flora and fauna [33], the development of analytical methods sensitive enough to determine them in this type of sample is one of the foci of attention of environmental research in recent decades [34–36].

The approach proposed in this article operates under the floating sampling technology mode, similarly to bar adsorptive microextraction (BAμE), introduced by Nogueira’s research group in 2010 [37]. In essence, this technique is based on the flotation of the sorptive phase in the sample, thanks to its lower density. Thus, the sorptive phase floats above the vortex caused by the stirring of a magnetic bar. Unlike previous modalities of the technique, in which the extraction phase was adhered to a cylindrical polyethylene or polypropylene tube using a double-sided adhesive tape, in the present work, the P-Ps/Ny-JP itself is used as the unique extraction device.

## Experimental

### Reagents

The following fourteen UV filters were selected as analytes: benzophenone-4 (BZ4) 99.9% from Roig Farma S.A. (Terrassa, Spain); benzophenone-3 (BZ3) 98% and ethylhexyl salicylate (EHS) 99% from Sigma-Aldrich (Barcelona, Spain, www.sigmaaldrich.com); isoamyl p-methoxycinnamate (IMC) 99.3% from Haarmann and Reimer (Parets del Vallés, Spain, www.symrise.com); phenylbenzimidazole sulfonic acid (PBSA) 99% and 4-methylbenzylidene camphor (MBC) 99.7% from Guinama S.L. (Valencia, Spain, www.guinama.com); octocrylene (OC) > 98% from F. Hoffmann-La Roche Ltd. (Basel, Switzerland, www.roche.com); ethylhexyl dimethyl PABA (EHDP) 100%, ethylhexyl methoxycinnamate (EHMC) 99.8%, and butyl methoxydibenzoylmethane (BMDM) 98% from Merck (Darmstadt, Germany, www.merckmillipore.com); diethylhexyl butamido triazone (DEBT) 99% from 3 V Iberia S.A. (Barcelona, Spain); ethylhexyltriazone (EHT) 100% and diethylamino hydroxybenzoyl hexyl benzoate (DHHB) 99.8% from BASF (Barcelona, Spain, www.basf.com/es); and drometrizole trisiloxane (DTS) 99.9% from L’Oréal (Paris, France). Their chemical structures and relevant information are given in Table S1. A stock solution containing 1000 μg mL^−1^ of both hydrophilic UV filters (i.e., BZ4 and PBSA) was prepared in deionized water. Likewise, a stock solution of the twelve lipophilic UV filters was prepared in methanol at an individual concentration of 1000 μg mL^−1^. Both stock solutions were stored at 4 °C in the dark. Working solutions to obtain the calibration plots were prepared by appropriate dilution in deionized water (Millipore Corp., Madrid, Spain) from a 500-ng mL^−1^ intermediate aqueous solution of all target analytes.

LC–MS grade methanol and LC–MS grade water from Panreac (Barcelona, Spain, www.itwreagents.com/iberia) were employed as solvents of the chromatographic mobile phase. Gradient-grade methanol from VWR chemicals (Fontenay-sous-Bois, France, es.vwr.com/store) was used as the elution solvent.

Common filter paper (grammage: 73 g m^−2^, thickness: 0.17 mm) from Filtros Anoia S.A. (Barcelona, Spain, fanoia.com), formic acid ≥ 99% from VWR chemicals, nylon-6 pellets from Sigma-Aldrich, polystyrene obtained from commercial yoghurt containers, and chloroform from Panreac (Barcelona, Spain) were used for the synthesis of the P-Ps/Ny-JP.

### Apparatus

Liquid chromatographic analyses were performed using an Agilent 1260 Infinity HPLC system (Agilent, Palo Alto, CA, USA) equipped with a binary high-pressure pump for mobile phase delivery and an autosampler. Identification and quantification of the analytes were performed on an Agilent 6420 triple quadrupole MS with electrospray source using the Agilent MassHunter Software (version B.06.00) for data analyses.

Magnetic stirrers from J.P. Selecta, S.A. (Barcelona, Spain) and a Vibramax 110 shaker from Heidolph Instruments (Schwabach, Germany) were used for the stirring during extraction and elution procedures, respectively.

Scanning electron microscope (SEM) images were obtained by using a JEOL JSM 7800F microscope (JEOL, Tokyo, Japan) at the Central Service for Research Support (SCAI) of the University of Córdoba. ATR-IR spectra were acquired with a Bruker Tensor 37 FT-IR spectrometer (Bruker Optik, GmbH, Ettlingen, Germany) equipped with a three internal reflections diamond ATR cell (Platinum ATR accessory, Bruker). Data collection and processing were done using the OPUS software package (Bruker, Ettlingen, Germany). Contact angle measurements were performed in a Ramé-hart Model 200 Standard Goniometer with DropImage Standard v2.3 equipped with an automated dispensing system at the Instituto de Ciencia Molecular (ICMol) of the University of Valencia.

### Sample preparation

Water samples were taken from two private swimming pools and Las Arenas Beach (Valencia, Spain), and they were analyzed as it will be described further on. All samples were collected in 2.5-L topaz glass bottles. Before analysis, unfiltered water samples were sonicated for 15 min in order to lixiviate the analytes from particles following the procedure previously developed to determine the total content of UV filters in the samples (i.e., the soluble fraction plus particulate fraction) [38]. Water samples were then conveniently diluted, if necessary, with deionized water to decrease the effect of ionic strength, as detailed later, in such a way the conductivity of the sample reached a value ≤ ca. 25 S cm^−1^.

### Synthesis of the paper-based polystyrene/nylon Janus platform

The Janus platform, based on a polystyrene/nylon-coated cellulosic sorptive phase, was prepared in a two-stage process. First, a filter paper segment (2 cm × 1 cm) was dipped halfway twice into 10 mL of a 6% w/v nylon-6 solution prepared in formic acid. After each dip, the solvent was completely evaporated at room temperature (ca. 10 min). Then, the other half was dipped twice into 10 mL of a 3% w/v polystyrene solution prepared in chloroform. As before, the solvent was evaporated at room temperature between each dip (ca. 5 min). The P-Ps/Ny-JP can be used for at least 2 weeks without loss of efficiency, and no additional cleaning step was required, as demonstrated in preliminary studies.

### Microextraction procedure

For the extraction step, a P-Ps/Ny-JP was introduced in a beaker containing 200 mL of the aqueous standard solution or water sample. Then, the solution was magnetically stirred at 1000 rpm for 30 min at room temperature. The extraction platform remained floating in the vortex generated by this stirring. After the extraction, the P-Ps/Ny-JP enriched with analytes was taken with clean tweezers, briefly rinsed by soaking it in deionized water, and dried at room temperature (ca. 5 min). Then, it was transferred to a 5-mL vial, in which 1 mL of methanol was added ensuring its total immersion to carry out the elution step. After 10 min of stirring in a shaker, the methanolic extract was placed into an injection vial for LC–MS/MS analysis. Considering the simplicity and low cost of the synthesis, the P-Ps/Ny-JP was discarded after each analysis.

### Liquid chromatography-tandem mass spectrometry analysis

Chromatographic separation was accomplished by reversed-phase separation on a Zorbax SB-C18 (50 × 2.1 mm i.d., 1.8 μm) column from Agilent. An in-line filter (0.2 μm, 2.1 mm i.d.), also from Agilent, was selected to preserve the integrity of the analytical column. The injection volume was 5 μL. The pumps supplied the following gradient at room temperature, being 0.1% aqueous formic acid (solvent A) and methanol (solvent B) the mobile phase: 0–1 min, 80% solvent B, 1–4 min linear gradient to 100% solvent B and held for 4 min. The flow rate was 0.3 mL min^−1^. The run time was 20 min.

Mass spectrometer settings were fixed to improve the multiple reaction monitoring (MRM) signals. The detector operated in positive electrospray ionization mode (ESI^+^) for lipophilic UV filters and in negative electrospray ionization mode (ESI^−^) for hydrophilic UV filters. The flow rate and the temperature of the drying gas (N_2_) were 11 L min^−1^ and 300 °C, respectively. The nebulizer pressure was 15 psi, and the capillary voltage was kept at 6000 V in both polarity modes. Table S2 summarizes the MRM parameters for the analytes. The most intense m/z ratio of the product ion (base peak) was selected as quantifier transition, and the second most intense product ion was selected as qualifier transition to ensure the identity of the analyte.

Figure S1 shows a chromatogram of an aqueous standard solution of the analytes at 500 ng L^−1^ subjected to the proposed method.

## Results and discussion

A plethora of different analytical methodologies have been used for the determination of UV filters in environmental waters involving solid-phase microextraction approaches. Among the multitude of extraction phases used for the extraction of these compounds, polydimethylsiloxane (PDMS) stands out both in solid-phase microextraction (SPME) and stir bar sorptive extraction (SBSE) approaches [35]. However, this phase is only suitable for UV filters of low to medium polarity. Efforts have been carried out to extract the most polar UV filters but in a lesser extent, using polyethyleneglycol-based phases [39] or a nylon-based magnetic material [40], among others. Therefore, the development of new devices that allow the determination of all types of UV filters, both hydrophobic and hydrophilic, is interesting and necessary.

### Evaluation of the paper-based polystyrene/nylon Janus platform

In order to evaluate the extraction ability and selectivity of the P-Ps/Ny-JP for a wide range of compounds of different polarities, fourteen UV filters with log *P* values from − 0.234 (PBSA) to 16.129 (EHT) were selected as model compounds (Table S1). It is important to note that even though all these compounds can be cataloged within the same application family (i.e., UV filters), they belong to different chemical groups, namely, benzophenone derivatives (BZ3, BZ4 and DHHB), p-aminobenzoic derivatives (EHDP), salicylates (EHS), methoxycinnamates (EHMC and IMC), camphor derivatives (MBC), triazine derivatives (EHT and DEBT), benzotriazole derivatives (DTS), benzimidazole derivatives (PBSA), and others (BMDM and OC). Additionally, among them, BZ4 and PBSA have a hydrophilic character and possess a negative charge in their structure due to the deprotonation of the sulfonate groups, whereas the remaining twelve exhibit lipophilic character. Therefore, and in terms of interaction chemistry with a sorbent phase, they cannot be jointly extracted with a high efficiency, as it is reported in the literature [35].

Firstly, the extraction capabilities of different cellulose-based supports for the extraction of all the analytes under study were compared. For this purpose, raw paper, nylon-coated paper (P-Ny), polystyrene-coated paper (P-Ps), and both polystyrene and nylon-coated paper (P-Ps/Ny) were used. These polymers were selected due to their affordability, easy preparation in solution, and expected interaction with analytes. In the synthesis of P-Ny and P-Ps supports, the paper (1 cm × 0.5 cm) was dipped in each polymer solution (i.e., nylon in formic acid, and polystyrene in chloroform) twice, alternatively changing the direction of immersion 180°. In the case of P-Ps/Ny support, each polymer covered half of the paper (2 cm × 1 cm). In all cases, the concentrations of the polymers were 3% (w/v).

The extraction efficiency (EE, %), calculated as the ratio of the amount of analyte extracted with respect to its initial amount in the donor phase, was used as response function. These experiments were accomplished by extracting 2 mL of aqueous standards at 50 ng mL^−1^ of the target analytes under non-optimized conditions. The stirring during extraction was maintained for 10 min at 1000 rpm. Elution of the analytes was performed with 1 mL of methanol, which was proven to be the best elution solvent in preliminary experiments, for 10 min. The employed volume was high enough to cover the entire support in a 5-mL vial.

**Fig. 1:**
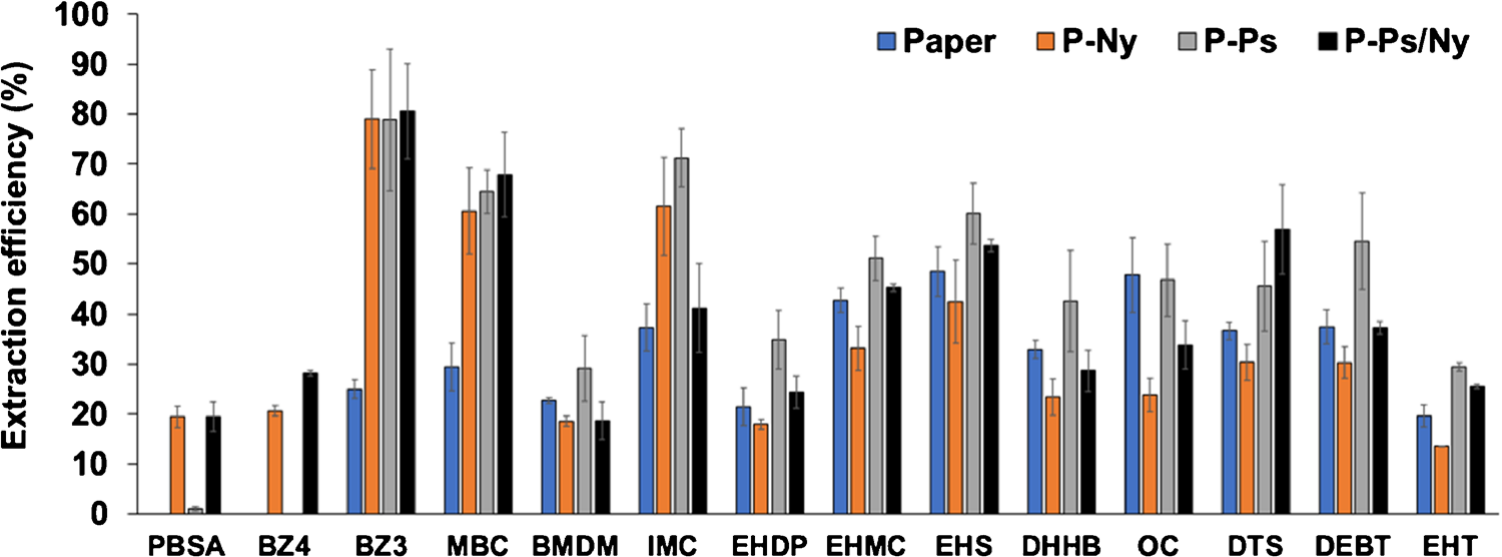
Comparison of extraction efficiencies of the analytes by using different extraction platforms (P-Ny, nylon-coated paper; P-Ps, polystyrene-coated paper; P-Ps/Ny, both polystyrene/nylon-coated paper) (*n* = 3)

As shown in the results depicted in Fig. 1, different trends were observed as a function of the polarity of the analytes. Thus, the two hydrophilic UV filters (i.e., PBSA and BZ4) were only extracted on P-Ny and P-Ps/Ny, probably due to the high affinity that sulfonated compounds have for polyamides such as nylon, as has been demonstrated in a previous work [40].

On the other hand, lipophilic compounds were, in general terms, more favorably extracted by using P-Ps support because of its hydrophobic nature, being this effect more pronounced when the polarity of the UV filters decreases. However, as mentioned above, the nylon was needed for the extraction of hydrophilic compounds, and therefore, the P-Ps support was discarded.

It should be mentioned that that the extraction through the P-Ps/Ny material allowed the extraction of all analytes, both hydrophilic and lipophilic, in a similar or even better way with respect to P-Ny. These results confirm the versatility and the complementary extraction of the P-Ps/Ny-JP for the simultaneous extraction of compounds of different polarity and chemical structures.

### Synthesis and characterization of the paper-based polystyrene/nylon Janus platform

Once the extraction efficiency and usefulness of the P-Ps/Ny-JP have been demonstrated, different factors that can affect the synthesis of the support were evaluated. In this sense, the coating procedure and the polymers proportion were studied.

**Fig. 2:**
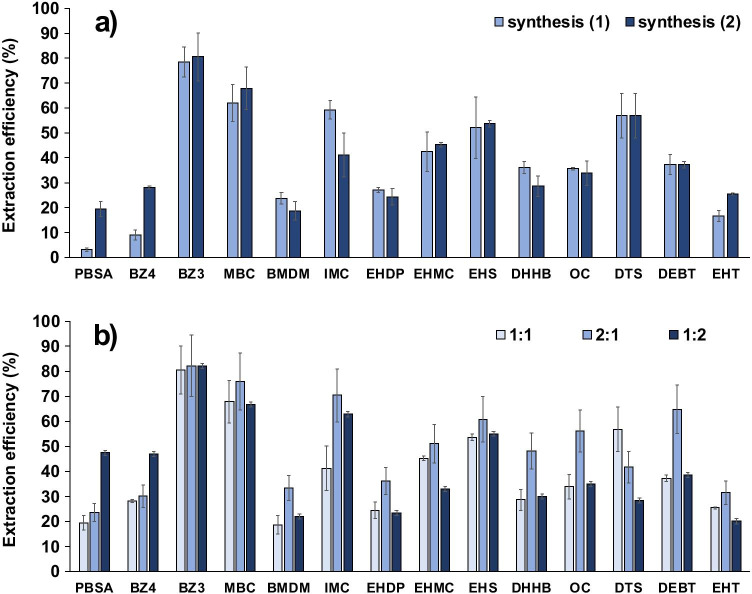
Comparison of extraction efficiencies for different **a** P-Ps/Ny-JP synthesis procedures (1, dipping the platform twice in a mixture of the polymers; 2, coating the platform with each polymer halfway) and **b** proportions of polystyrene and nylon, respectively, in the synthesis of the platforms

First, it was compared (1) dipping the paper (2 × 1 cm) twice in a mixture of the two polymers (3% w/v) prepared in their respective solvents (i.e., nylon in formic acid and polystyrene in chloroform); and (2) coating the paper in halves (i.e., each polymer in one half of the paper), being both polymers at concentrations of 3% (w/v). In the former case, the mixture was not homogeneous due to the immiscibility of the solvents. Therefore, it was necessary to shake the mixture before each dipping. The same extraction conditions described in the previous section were used. As can be seen in Fig. 2a, no significant differences were observed except for the two hydrophilic compounds (extracted by nylon), for which a better response was obtained when coating the paper in halves. This may be due to the fact that when the two polymers were previously mixed, the polystyrene also overlapped part of the nylon on the paper, thus preventing the interaction of these two compounds with the nylon. Thus, coating the paper in halves was adopted for the following studies.

Regarding the proportion of polymers, 1:1, 1:2 and 2:1 (polystyrene:nylon, Ps:Ny) were evaluated. The solutions of the polymers were prepared either at 3% (w/v) or at 6% (w/v) depending on the desired proportion of each polymer. Thus, the paper was always dipped in each polymer twice and the number of dips did not influence the extraction efficiency of the Janus platform. According to the results depicted in Fig. 2b, two trends were observed depending on the nature of the analytes. On the one hand, for those analytes extracted by nylon, better responses were obtained when the 1:2 ratio was used, and on the other hand, most lipophilic compounds were better extracted when the 2:1 ratio was used. To favor the extraction of the two hydrophilic compounds, the 1:2 Ps/Ny ratio was selected as optimal for the synthesis of the P-Ps/Ny-JP.

**Fig. 3:**
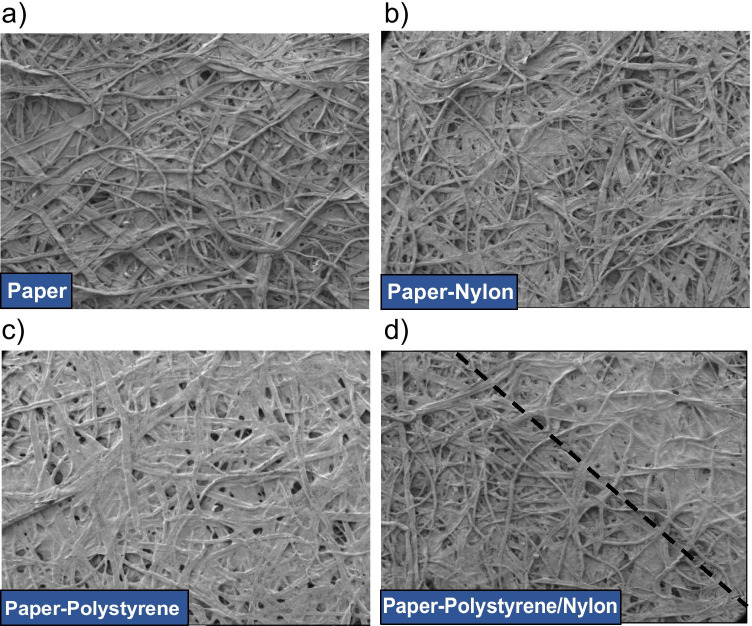
SEM images of **a** raw paper, **b** nylon-coated paper, **c** polystyrene-coated paper, and **d** interface of polystyrene/nylon in the P-Ps/Ny-JP, at magnification 60

The Janus substrate was analyzed by SEM to study its morphology. As shown in Fig. 3a, the paper is composed of a non-uniform three-dimensional network of cellulosic fibers. Figure 3b shows the partial coating of the paper surface by the nylon making it rougher, whereas the polystyrene is deposited on the paper more homogeneously, hiding the cellulose fibers of the paper (Fig. 3c). Finally, a detailed observation of the interface between nylon and polystyrene in the P-Ps/Ny-JP is shown in Fig. 3d, where both phases are clearly differentiated (i.e., the upper one is polystyrene and the lower one corresponds to nylon).

Likewise, the Janus material was also analyzed by ATR-IR (Fig. S2). Nylon has characteristic and well-defined bands at 1537 cm^−1^ (NH deformation) and at 1633 cm^−1^ (C = O stretching). The main bands of paper are found at 2897 cm^−1^ (C-H symmetrical stretching), 3336 cm^−1^ (O–H stretching), and strong absorption in the range of 1033 cm^−1^ (C-O stretching), whereas polystyrene possesses defined bands at 694 and 746 cm^−1^ (C-H bending), and at 1450 and 1491 cm^−1^ (C–C stretching vibrations). In Fig. S2d and S2e, the characteristics peaks of paper and nylon and paper and polystyrene, respectively, are observed. The IR spectrum of the interface between nylon and polystyrene in the platform is presented in Fig. S2f, in which all the characteristic peaks and bands are observed, and thus indicating the success in the synthesis.

Finally, the contact angle of water on both parts of the support (i.e., nylon and polystyrene) was measured to show their different hydrophobicity. In the case of polystyrene side, an angle of 97.5° was obtained (Fig. S3a), demonstrating its lipophilic character, whereas in the nylon side, the water drop was not maintained on the surface due to its hydrophilic character (Fig. S3b). Figure S3c shows a photograph of P-Ps/Ny-JP with a drop of water on each polymer.

### Optimization of the microextraction procedure using the paper-based polystyrene/nylon Janus platform

Several critical variables (namely, sample volume, extraction time, elution time, pH, and ionic strength) were considered in the optimization of the microextraction procedure. A univariate methodology was followed to understand the separate influence of each variable. In the initial conditions (i.e., non-optimized ones), aqueous standard solutions containing the fourteen UV filters at 50 ng mL^−1^ were extracted for 10 min and the analytes were eluted with 1 mL of methanol for 10 min. The P-Ps/Ny-JP was synthesized as aforementioned. The enrichment factor (EF), determined as the ratio between the response of the aqueous standard solution after the extraction and the response obtained with the direct injection of this standard solution without extraction, was used as response function.

The results obtained for the optimization of sample volume, extraction time, elution time, and pH are included in the Supplementary Information (Fig. S4).

The effect of the ionic strength of the donor phase was assessed using sodium chloride as model electrolyte within the concentration range 0–10% (w/v). These concentrations are equivalent to conductivity values from 0 to 160 S cm^−1^. The EFs decreased for all analytes, being more pronounced for values higher than 2% (w/v), which can be ascribed to an increase of the water viscosity, thus hindering the transference of the analytes from the bulk sample to the extraction support. This fact can cause problems when seawater samples are analyzed, since their salt content can reach up 3% (w/v). In order to prevent this problem, the dilution of the sample to reduce the influence of this variable on the recovery of the analytes was evaluated. As can be seen in Fig. S5, when dilution of an aqueous standard solution adjusted to 3% (NaCl, w/v, conductivity ca. 48 S cm^−1^) was diluted with deionized water at 1:1 ratio (thus resulting in 1.5% (w/v) of NaCl content), the EFs increased up to values similar to a standard without adjusting the ionic strength. Therefore, sample dilution of 1:1 was adopted for further analysis. These results showed that the conductivity should be measured, evaluated, and eventually reduced by sample dilution until it reaches a value ≤ ca. 25 S cm^−1^ when working with real samples, since the natural ionic strength of the sample may decrease the extraction of the analytes.

### Analytical figures of merit of the proposed method

Once the variables that affect the extraction were selected, the main quality parameters of the proposed method were evaluated, namely, linearity, enrichment factors, limits of detection (LOD) and quantification (LOQ), and precision. The figures of merit are compiled in Table 1.

**Table 1:** Main quality parameters of the proposed method

UV filter	EF^a^	EE^a^ (%)	LOD^b^ (ng L^−1^)	LOQ^b^ (ng L^−1^)	Repeatability (% RSD)^c^				
					Intra-day		Inter-day		
					500 ng L^−1^	1000 ng L^−1^	500 ng L^−1^		1000 ng L^−1^
PBSA	82	41	28	93	12.7	7.8	14.7	12.3	
BZ4	36	18	56	185	13.1	9.7	8.1	11.1	
BZ3	64	32	20	67	10.2	10.1	10.7	12.6	
MBC	54	27	29	98	8.1	6.1	7.3	11.1	
BMDM	86	43	20	68	9.2	8.2	8.8	8.0	
IMC	75	37	28	93	4.7	12.5	5.0	12.8	
EHDP	61	30	22	74	5.3	12.2	7.5	9.6	
EHMC	125	62	12	41	9.3	7.2	12.2	9.7	
EHS	31	16	60	200	10.4	9.6	8.1	8.6	
DHHB	110	55	22	75	13.0	8.8	11.1	13.9	
OC	122	61	18	59	10.8	10.5	8.3	11.6	
DTS	31	16	71	238	7.9	5.3	9.1	6.2	
DEBT	95	48	26	86	5.8	2.7	7.6	8.9	
EHT	45	22	27	91	9.0	10.4	11.9	11.5	

^a^*EF* enrichment factor; *EE* extraction efficiency

^b^*LOD* limit of detection; *LOQ* limit of quantification; calculated as 3 times and 10 times, respectively, the signal-to-noise ratio

^c^*RSD* relative standard deviation

The linear range was evaluated using aqueous standard solutions at six concentration levels from 0.3 to 100 ng mL^−1^ for three UV filters (i.e., BZ4, EHS, and DTS) and from 0.1 to 100 ng mL^−1^ for the others UV filters, which were submitted to the microextraction procedure. Due to the low concentrations of these compounds expected in real water samples, the working calibration range was set from 100 or 300 ng L^−1^, depending on the analyte, to 1000 ng L^−1^, with R^2^ ≥ 0.994 in all cases. The estimated EFs were between 31 and 125, which corresponds to extraction efficiencies (EE) (calculated considering an EF_max_ of 200) between 16 and 62%. The LODs and LOQs were calculated as the concentration corresponding to a value of signal-to-noise of 3 and 10, respectively. These values were found to be in the ranges 12–71 ng L^−1^ and 41–238 ng L^−1^, respectively, which agree with the EFs obtained. The precision of the method was evaluated as relative standard deviation (RSD, %) from the analysis of five different aqueous standard solutions containing the target analytes at 500 and 1000 ng L^−1^ in the same day (intra-day repeatability) and in five different working sessions (inter-day repeatability). These values were between 4.7 and 14.7%, thus showing the good precision of the method. The reproducibility of five P-Ps/Ny-JP prepared in different sessions was also studied. In this case, RSDs < 9.6% were obtained, thus demonstrating that the macroscopic variability that may exist between the different supports does not affect the repeatability of the extraction, and therefore the practical applicability is assured.

### Application to the analysis of water samples

As a proof-of-concept of the P-Ps/Ny-JP, it was applied to the analysis of real water samples likely to contain UV filters: two private swimming pool water and one seawater. Furthermore, it should be noted that the salinity of these three samples was different, since one of the pools was treated water (swimming pool 1), and the other was saline (swimming pool 2). As previously mentioned, the conductivity of the samples was measured to estimate their saline content. Thus, the conductivity values for the two swimming pool water samples were 1.4 and 4.3 S cm^−1^, respectively (which correspond to approximate values of 0.08 and 0.2% NaCl (w/v)), while for the sea water sample, the conductivity value was 45.7 S cm^−1^ (ca. 2.9% of salt content). For this reason, the first two samples were analyzed without prior treatment, while the seawater sample was diluted with deionized water at ratio 1:1 prior to analysis (conductivity ca. 23 S cm^−1^). The samples were analyzed in triplicate according to the developed method. As can be seen in Table 2, all samples presented quantities of the target UV filters, predictably due to the use of sunscreens by bathers in these samples. It should be noted that the concentrations of these compounds, especially in seawater samples, depend on various factors such as the moment of sampling or the water tide, among others [41].

**Table 2:** UV filters’ contents in three water samples obtained by applying the proposed method (*n* = 3)

UV filter	Found amount (ng L^−1^)		
	Swimming pool 1	Swimming pool 2	Sea
PBSA	< LOD	179 ± 2	< LOD
BZ4	540 ± 40	< LOD	< LOD
BZ3	< LOD	480 ± 30	704 ± 70
MBC	< LOD	< LOD	< LOD
BMDM	281 ± 2	217 ± 10	815 ± 80
IMC	< LOD	< LOD	< LOD
EHDP	< LOD	< LOD	< LOD
EHMC	< LOD	361 ± 9	595 ± 60
EHS	750 ± 40	< LOD	600 ± 20
DHHB	< LOD	210 ± 10	487 ± 20
OC	300 ± 3	335 ± 7	< LOD
DTS	< LOD	< LOQ	< LOD
DEBT	205 ± 3	137 ± 10	< LOD
EHT	< LOD	< LOD	< LOD

Finally, the reliability of the proposed method and the presence of matrix effects were evaluated by recovery studies. For this purpose, the three above-mentioned water samples were spiked with the target analytes at 500 and 1000 ng L^−1^, and subjected to the extraction process. The results presented in Table 3 shows that the obtained relative recoveries ranged from 81 to 121% in swimming pool waters and from 73 to 101% in sea water, thus demonstrating the absence of matrix effects, beyond the effect of ionic strength in the seawater sample already considered and corrected by sample dilution.

**Table 3:** Relative recoveries values obtained by applying the proposed method to three water samples spiked at two concentration level concentration (*n* = 3)

UV filter	Swimming pool 1		Swimming pool 2		Sea	
	500 ng L^−1^	1000 ng L^−1^	500 ng L^−1^	1000 ng L^−1^	500 ng L^−1^	1000 ng L^−1^
PBSA	114 ± 10	119 ± 3	120 ± 7	114 ± 10	81 ± 2	90 ± 7
BZ4	106 ± 10	98 ± 2	98 ± 10	81 ± 2	86 ± 10	81 ± 2
BZ3	118 ± 10	107 ± 5	96 ± 8	83 ± 2	96 ± 10	84 ± 2
MBC	105 ± 5	111 ± 7	103 ± 10	112 ± 10	86 ± 9	77 ± 10
BMDM	105 ± 10	88 ± 4	115 ± 5	103 ± 7	99 ± 5	94 ± 7
IMC	108 ± 10	94 ± 10	114 ± 10	103 ± 7	92 ± 9	82 ± 6
EHDP	97 ± 9	114 ± 3	97 ± 10	113 ± 2	77 ± 8	90 ± 2
EHMC	94 ± 6	119 ± 5	104 ± 10	84 ± 2	87 ± 10	82 ± 2
EHS	108 ± 10	100 ± 10	84 ± 8	86 ± 3	84 ± 20	86 ± 2
DHHB	96 ± 10	119 ± 2	115 ± 2	115 ± 5	101 ± 4	80 ± 4
OC	97 ± 2	100 ± 7	101 ± 2	107 ± 10	95 ± 2	92 ± 3
DTS	109 ± 2	113 ± 6	83 ± 5	98 ± 8	79 ± 5	96 ± 8
DEBT	100 ± 4	98 ± 2	105 ± 5	121 ± 3	92 ± 2	80 ± 2
EHT	116 ± 10	97 ± 9	109 ± 8	114 ± 7	73 ± 5	76 ± 5

### Comparison of the proposed method with other alternatives for determination of UV filters in waters

The main analytical features of the proposed method were compared with other common microextraction approaches previously reported with the same purpose, covering the largest number of UV filters in common with this work (Table 4). Although the lowest LODs were not obtained by this method, they were in the low ng L^−1^ range, low enough to determine these compounds in this type of matrix, as demonstrated in the previous section. In any case, if necessary, the method can include a later step of evaporation of the elution solvent and subsequent reconstitution in a smaller volume, in order to further reduce the LOQs. Regarding accuracy, all studies presented relative recovery values higher than 70%.

**Table 4:** Comparison of the proposed method with other microextraction approaches for determination of UV filters in waters

Number of UV filters^a^	Extraction technique^b^	Extraction phase^c^	Analytical technique^d^	Sample volume (mL)	Extraction time (min)	Comments	EF	LOD (ng L^−1^)	Relative recovery (%)	Ref
Lipophilic										
9: BMDM, BZ3, EHDP, EHMC, EHS, HMS, IMC, MBC, OR	SBSE	PDMS-coated stir bar	TD-GC–MS	20	180	-	n.r	0.2–63	75–116	[42]
8: BZ3, MBC, IMC, EHDP, EHMC, EHS, HMS, OC	DLLME	Chloroform	GC–MS	5	-	-	112–263	10–30	82–117	[38]
8: BZ3, MBC, IMC, EHDP, EHMC, EHS, HMS, OC	SBSDME	CoFe_2_O_4_@oleic acid MNPs	TD-GC–MS	25	30	Evaporation and reconstitution	68–690	13–148	80–116	[43]
Hydrophilic										
4: BZ4, PBSA, PDTA, TDSA	SBSDME	CoFe_2_O_4_@SiO_2_-nylon 6 composite	LC-UV	25	30	Evaporation and reconstitution	105–145	1600–2900	90–115	[40]
Lipophilic and hydrophilic										
14: BS, BZ1, BZ3, BZ4, BZ8, EHDP, EHMC, EHS, Eto, HMS, IMC, MA, MBC, OC	(DI)SPME	DVB/CAR/PDMS-coated fiber	TD-GC–MS/MS	10	30	In situ derivatization	n.r	0.045–8.2	80–106	[44]
14: BZ3, BZ4, BMDM, DEBT, DHHB, DTS EHDP, EHMC, EHS, EHT, IMC, MBC, OC, PBSA	-	P-Ps/Ny Janus platform	LC–MS/MS	200	30	-	31–125	12–71	73–121	This work

^a^*BMDM* butyl methoxydibenzoylmethane; *BZ3* benzophenone-3; *BZ4* benzophenone-4; *DEBT* diethylhexyl butamido triazone; *DHHB* diethylamino hydroxybenzoyl hexyl benzoate; *DTS* drometrizole trisiloxane; *EHDP* thylhexyl dimethyl PABA; *EHMC* 2-ethylhexyl 4-methoxycinnamate; *EHS* 2-ethylhexyl salicylate; *EHT* ethylhexyl triazone; *IMC* isoamyl 4-methoxycinnamate; *MBC* 3-(4-methylbenzylidene)camphor; *OC* octocrylene; *PBSA* 2-phenylbenzimidazole-5-sulfonic acid

^b^*(DI)SPME* direct-immersion solid-phase microextraction; *DLLME* dispersive liquid–liquid extraction; *SBSDME* stir bar sorptive dispersive microextraction; *SBSE* stir bar sorptive extraction

^c^*DVB/CAR/PDMS* divinylbenzene-carboxen-polydimethylsiloxane; *MNPs* magnetic nanoparticles; *PDMS* polydimethylsiloxane; *P-Ps/Ny* paper-based polystyrene/nylon

^d^*GC* gas chromatography; *LC* liquid chromatography; *MS* mass spectrometry; *MS/MS* tandem mass spectrometry; *TD* thermal desorption; *UV* ultraviolet

Nevertheless, the main advantages of the proposed P-Ps/Ny-JP that deserve to be highlighted are the sustainability, simplicity in synthesis, and low cost of this extraction device. The starting materials used to prepare it (i.e., common filter paper, nylon, and polystyrene, which can even come from commercial packaging as in this article) are purchased in bulk, which is very competitive compared with SPME fibers or SBSE units subjected to commercial patents. In addition, the combination of different polymers offered a considerable improvement in the versatility and selectivity with respect to most approaches, which are mainly focused on the determination of lipophilic UV filters, in a simple way, allowing the simultaneous determination of a large number of UV filters, both hydrophilic and lipophilic. Additionally, no conditioning, derivatization or centrifugation steps are required in the proposed methodology, and no organochloride solvents are used during extraction procedure but only in the preparation of the support.

The main drawback of the proposed support is the independent and manual synthesis of each one, but remediable by automating the dip coating process, as well as the possibility of physical variation of the supports during synthesis, although it was shown that it did not significantly affect the repeatability in the extraction.

## Conclusions

In the present article, a new and easy-to-prepare mix-mode cellulose-based sorptive phase with both hydrophilic and hydrophobic domains is presented. This Janus material is prepared using two affordable polymers such as nylon and polystyrene. The preparation of the extraction device only requires dipping the paper into organic solutions containing both polymers, and exhibits significant advantages, such as low cost and simple manipulation. The analytical potential of this new material is evaluated by the extraction of fourteen UV filters with very different polarity from waters. Although the polarity index (expressed as log P_ow_ values) ranged from − 0.234 to 16.129, the absolute extraction efficiencies varied between 16 and 62%. These values, which are good for a microextraction procedure, provide enrichment factors in the interval 31–125 increasing the sensitivity of the analytical method. The advantage of being able to determine these UV filters of different polarity with different chemical structures by this Janus platform opens up new applications to extract many other families of compounds in a sustainable and economical way.

## Supplementary Information

Below is the link to the electronic supplementary material.

Supplementary file1 (DOCX 3317 KB)
