# Supplementary material for: A paper-based polystyrene/nylon Janus platform for the microextraction of UV filters in water samples as proof-of-concept
Source: Mikrochim Acta. 2021 Oct 25;188(11):391. doi: 10.1007/s00604-021-05047-x (PMC8542539; doi:10.1007/s00604-021-05047-x)
Supplement: Supplementary file 1 — Supplementary file1 (DOCX 3317 KB) [file 604_2021_5047_MOESM1_ESM.docx]

**Electronic Supplementary Material**

**A paper-based polystyrene/nylon Janus-platform for the microextraction of UV filters in water samples as proof-of-concept**

**Juan L. Benedé,^a,b^ Alberto Chisvert,^b^ Rafael Lucena,^a^ and Soledad Cárdenas^a^**

^a^ Departamento de Química Analítica, Instituto Universitario de Investigación en Química Fina y Nanoquímica IUNAN, Universidad de Córdoba, Campus de Rabanales, Edificio Marie Curie (anexo), E-14071, Córdoba, Spain

^b^ Department of Analytical Chemistry, University of Valencia, 46100 Burjassot, Valencia, Spain

**TABLE OF CONTENTS**

**Fig. S1.** Chromatograms of an aqueous standard solution of the analytes at 500 ng L^-1^ after applying the proposed method1

**Fig. S2.** ATR-IR spectra of (a) nylon, (b) raw paper, (c) polystyrene, (d) paper-nylon side, (e) paper-polystyrene side, and (f) interface of polystyrene/nylon in the Ps/Ny Janus-paper.2

**Fig. S3.** Contact angle measurement of polystyrene side in the P-Ps/Ny-JP3

Optimization of the microextraction procedure using the paper-based polystyrene/nylon Janus-platform4

**Fig. S4.** Effect of the (a) sample volume, (b) extraction time, and (c) desorption time on the enrichment factors of the analytes.5

**Fig. S5.** Effect of sample dilution on the enrichment factors of the analytes. The ionic strength is indicated in brackets.6

**Table S1.** Chemical structure and some relevant data of the target compounds7

**Table S2.** Tandem mass spectrometry parameters for the target compounds9

**Fig. S1**. Chromatograms of an aqueous standard solution of the analytes at 500 ng L^-1^ after applying the proposed method.

**Fig. S2**. ATR-IR spectra of (a) nylon, (b) raw paper, (c) polystyrene, (d) paper-nylon side, (e) paper-polystyrene side, and (f) interface of polystyrene/nylon in the Ps/Ny Janus-paper.


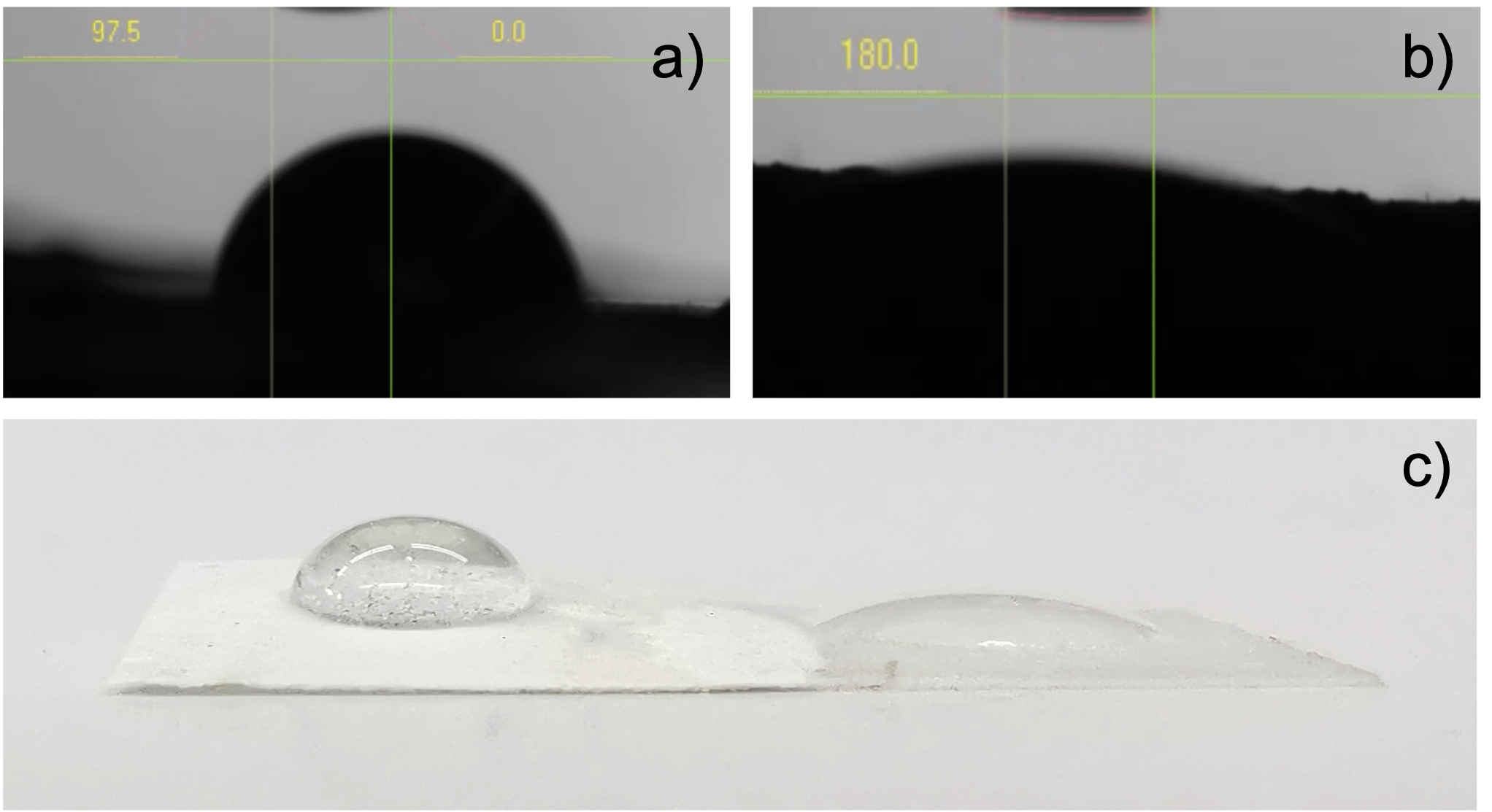


**Fig. S3**. (a) Contact angle measurement of polystyrene side in the P-Ps/Ny-JP; (b) contact angle measurement of polystyrene side in the P-Ps/Ny-JP; (c) photograph of P-Ps/Ny-JP with a drop of water on each polymer.

**Optimization of the microextraction procedure using the paper-based polystyrene/nylon Janus-platform**

The sample volume was the first variable considered and it was studied within the interval 20-200 mL. The profiles for all compounds (Fig. S1a) showed that EFs increased considerably with increasing sample volume up to 200 mL as was expected. Higher volumes were not tested due to poor stability of the P-Ps/Ny-JP at the vortex during stirring, which would have resulted in worse data reproducibility.

The effect of extraction time on the EFs was investigated in the range 5-60 min. The optimum extraction was accomplished within 20-30 min, depending on the analytes (Fig. S1b). In order to favor the extraction of the two hydrophilic compounds (i.e., BZ4 and PBSA), 30 min was finally selected for further experiments.

The elution time profiles of the analytes were assayed at different values (5-30 min). The results depicted in Fig. S1c showed that the EFs increased from 5 to 10 min for most analytes, with a negligible increase for longer times. In fact, the signal decreases for some analytes at long times. Thus, 10 min was adopted as optimal. The relative amount of analytes measured in a second desorption on the same P-Ps/Ny-JP was less than 3.7 %, thus indicating that no additional elution steps are necessary.

The influence of the pH of the donor aqueous solution was studied in the range 2–8. No significant differences were observed in terms of the analytical signal, so the pH was not adjusted in subsequent experiments.

**a)**

**b)**

**c)**

**Fig. S4.** Effect of the (a) sample volume, (b) extraction time, and (c) desorption time on the enrichment factors of the analytes.

**Fig. S5**. Effect of sample dilution on the enrichment factors of the analytes. The ionic strength is indicated in brackets.

**Table S1.** Chemical structure and some relevant data of the target compounds.

| UV filter | Chemical structure | CAS | logP_ow_ ^a^ | pK_a_ ^b^ |
| --- | --- | --- | --- | --- |
| 2-Phenylbenzimidazole-5-sulfonic acid  (PBSA) | 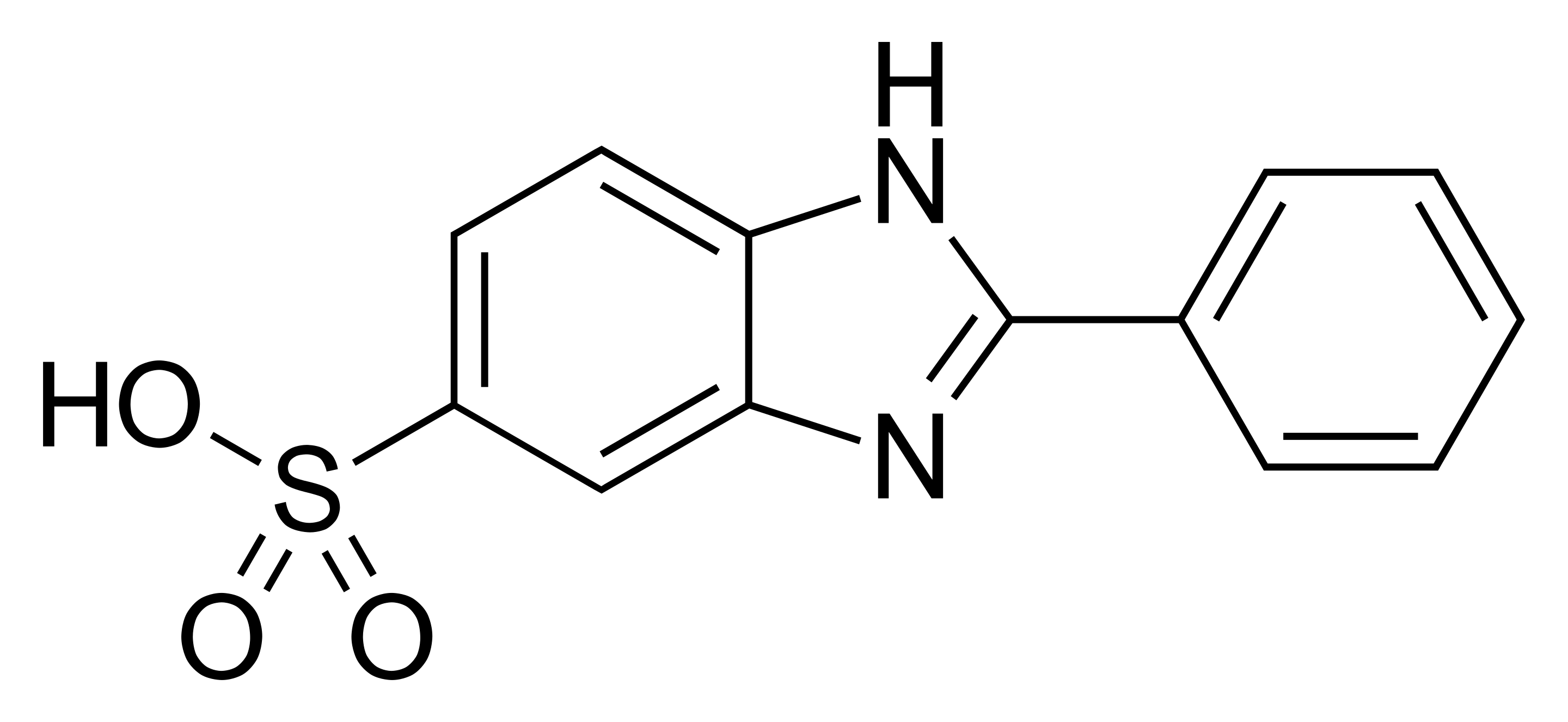 | 27503-81-7 | -0.234 | -0.87 (a) 4.16 (b) |
| Benzophenone-4  (BZ4) | 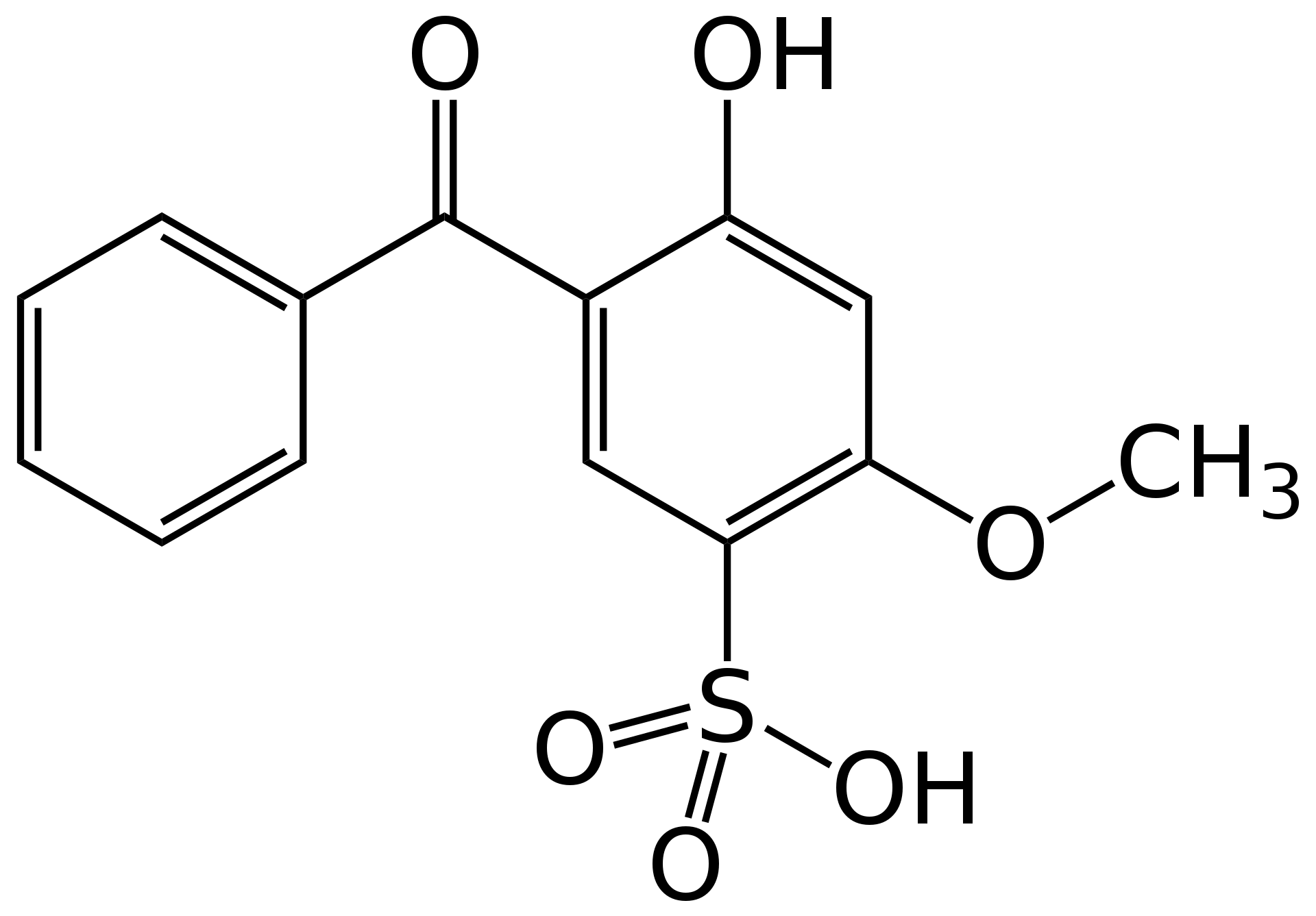 | 4065-45-6 | 0.993 | -0.70 |
| Benzophenone-3  (BZ3) | 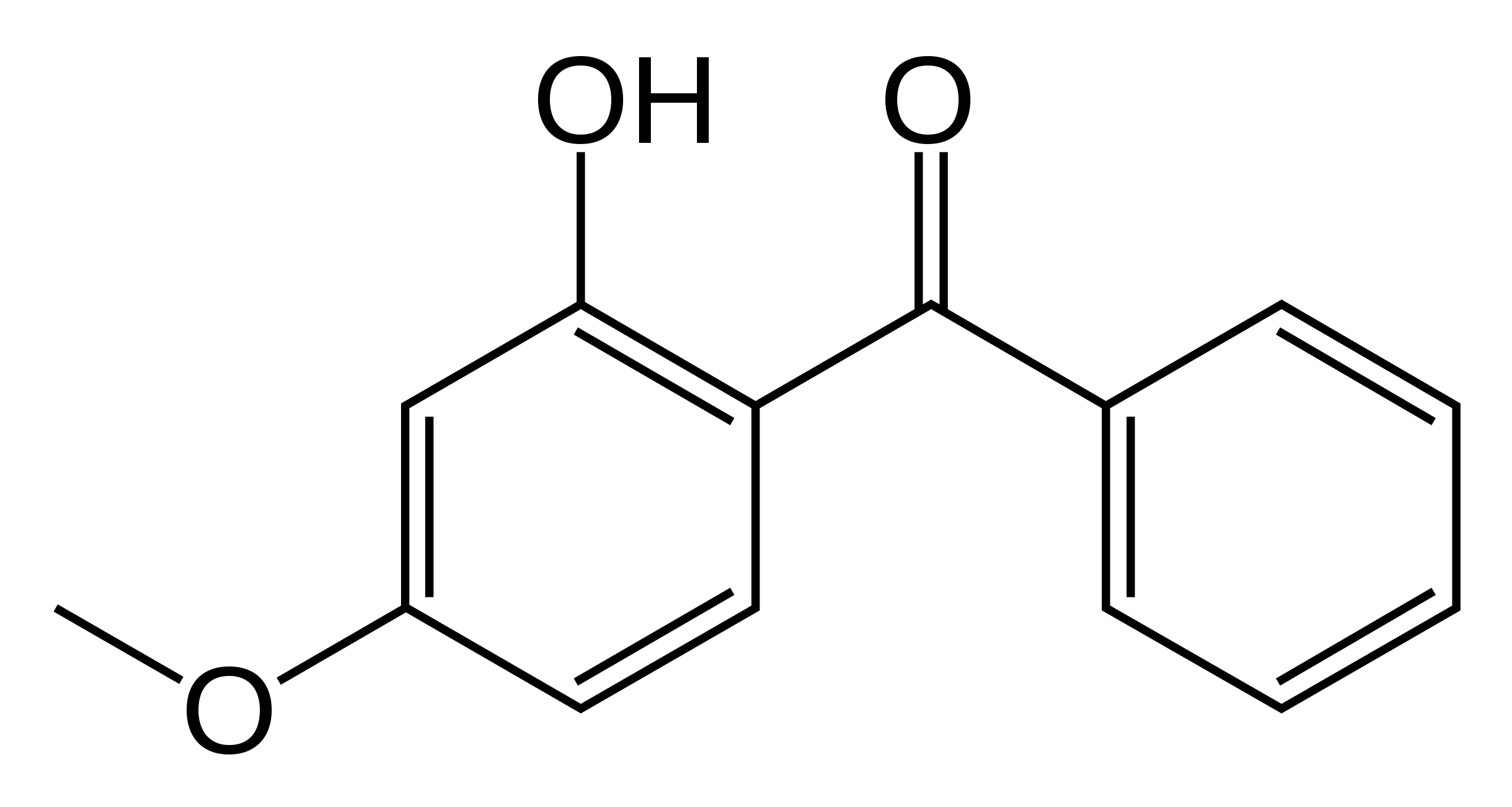 | 131-57-7 | 3.995 | 7.56 |
| 3-(4-methylbenzylidene)camphor  (MBC) | 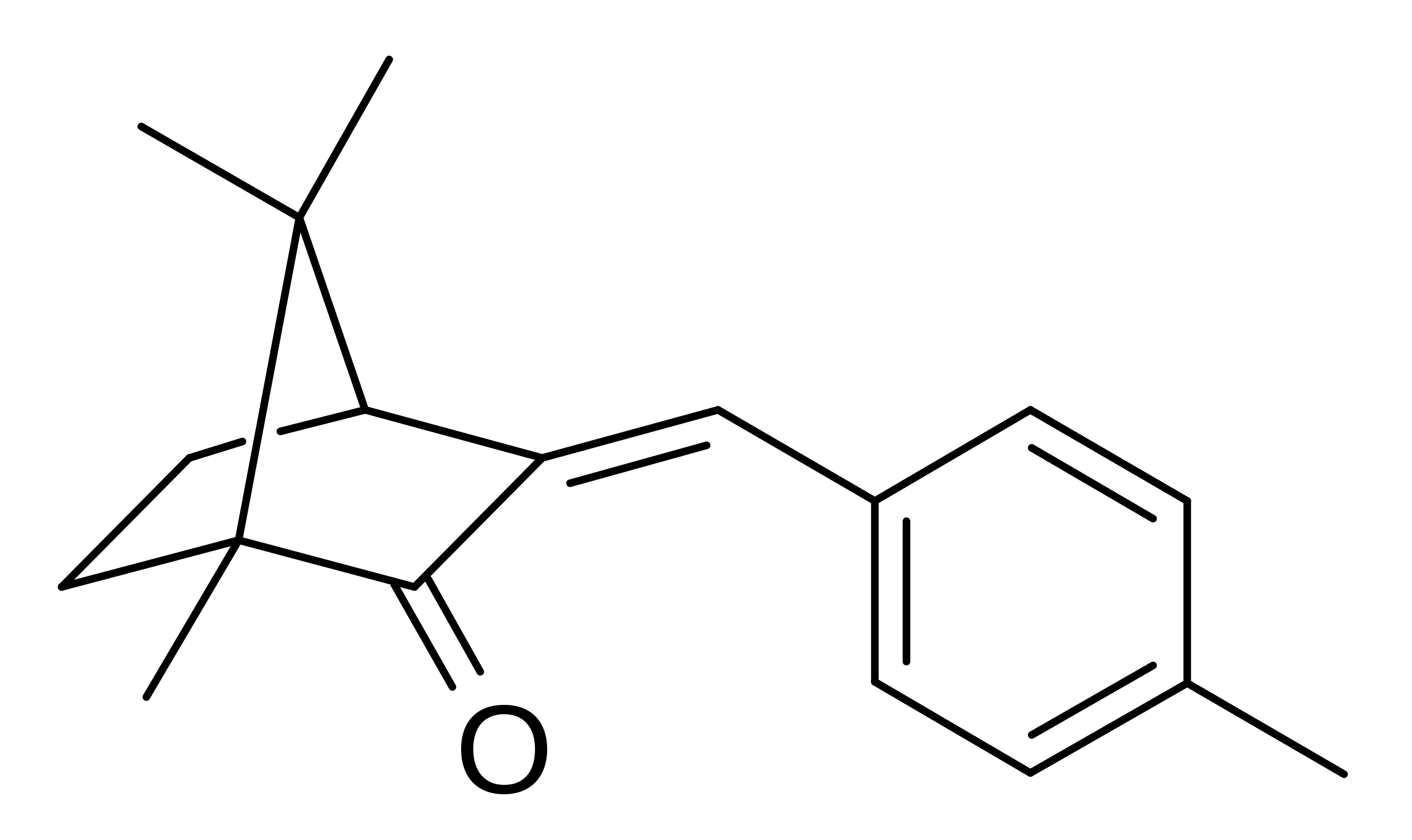 | 36861-47-9 | 3.385 | - |
| Butyl methoxydibenzoyl methane (BMDM) | 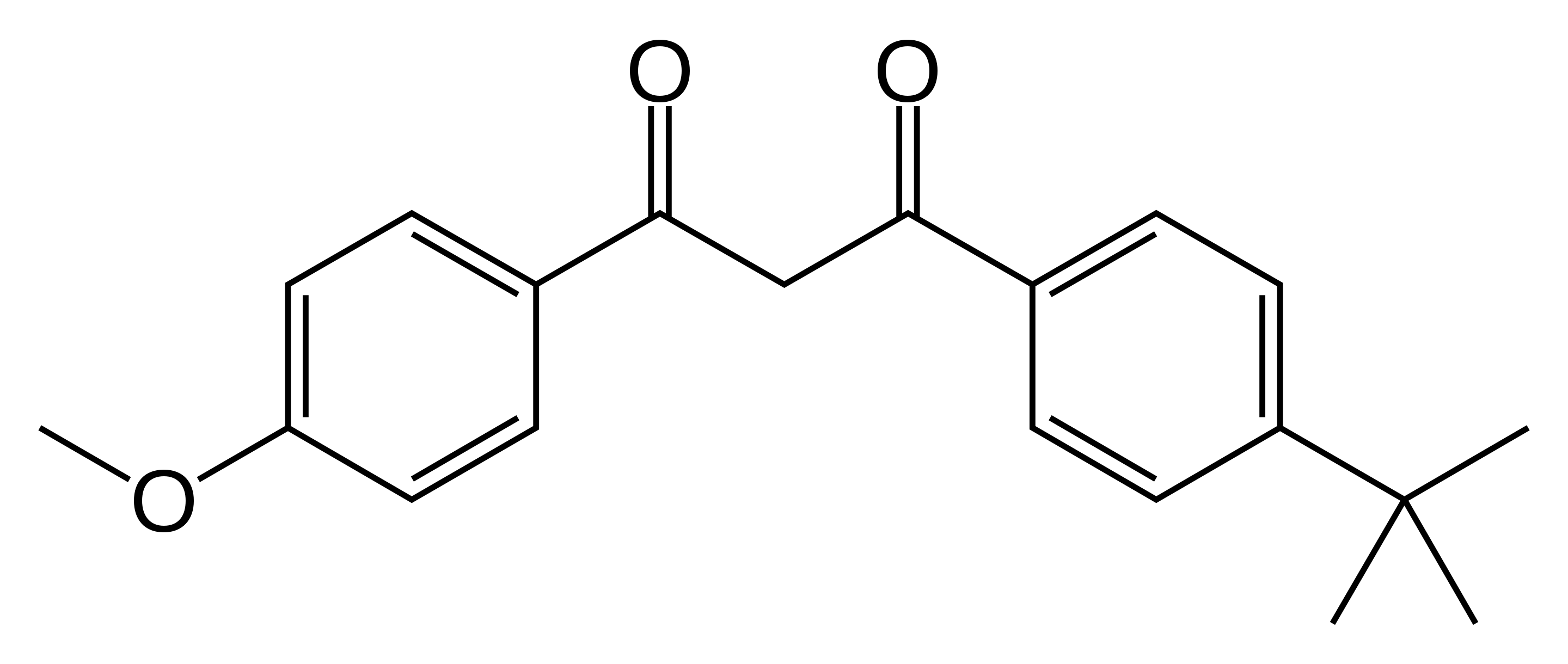 | 70356-09-1 | 4.191 | 9.74 |
| Isoamyl 4-methoxycinnamate  (IMC) | 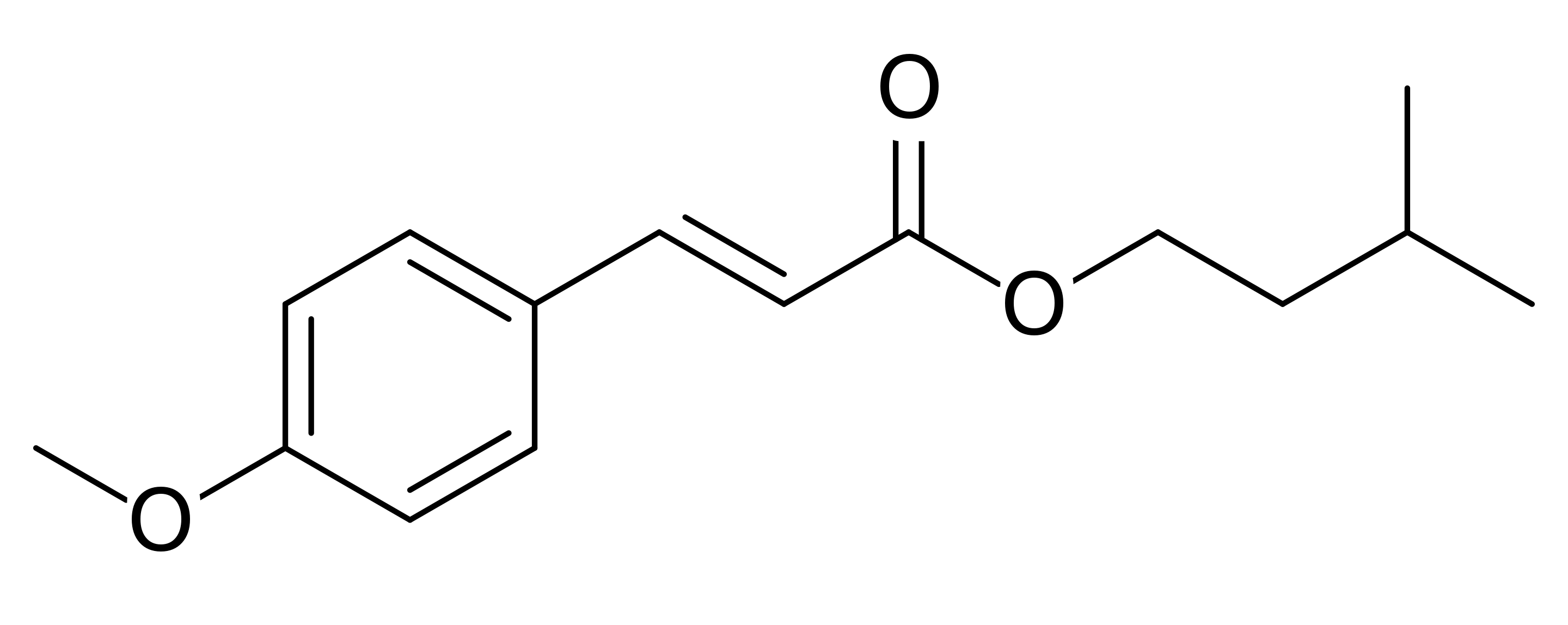 | 71617-10-2 | 4.393 | - |
| Ethylhexyl dimethyl PABA  (EHDP) | 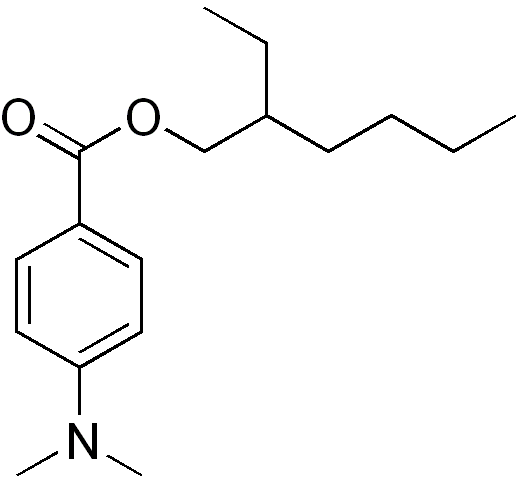 | 21245-02-3 | 5.412 | 2.39 (b) |
| 2-ethylhexyl 4-methoxycinnamate (EHMC) | 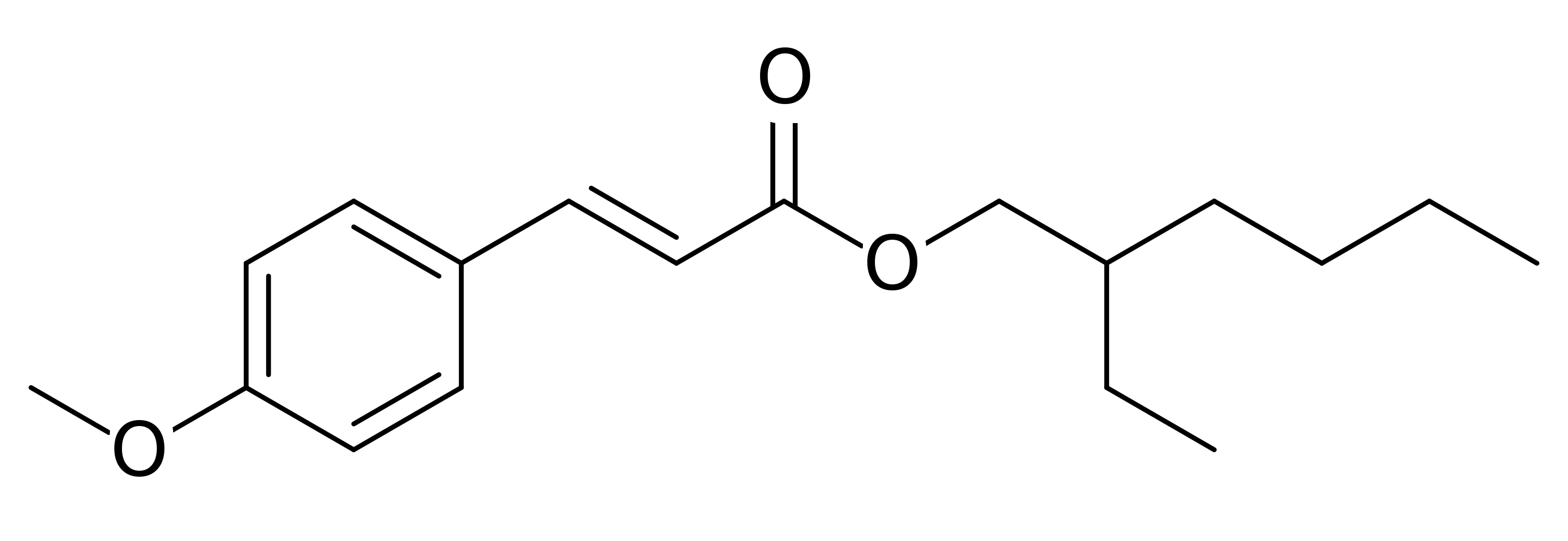 | 5466-77-3 | 5.921 | - |
| 2-ethylhexyl salicylate  (EHS) | 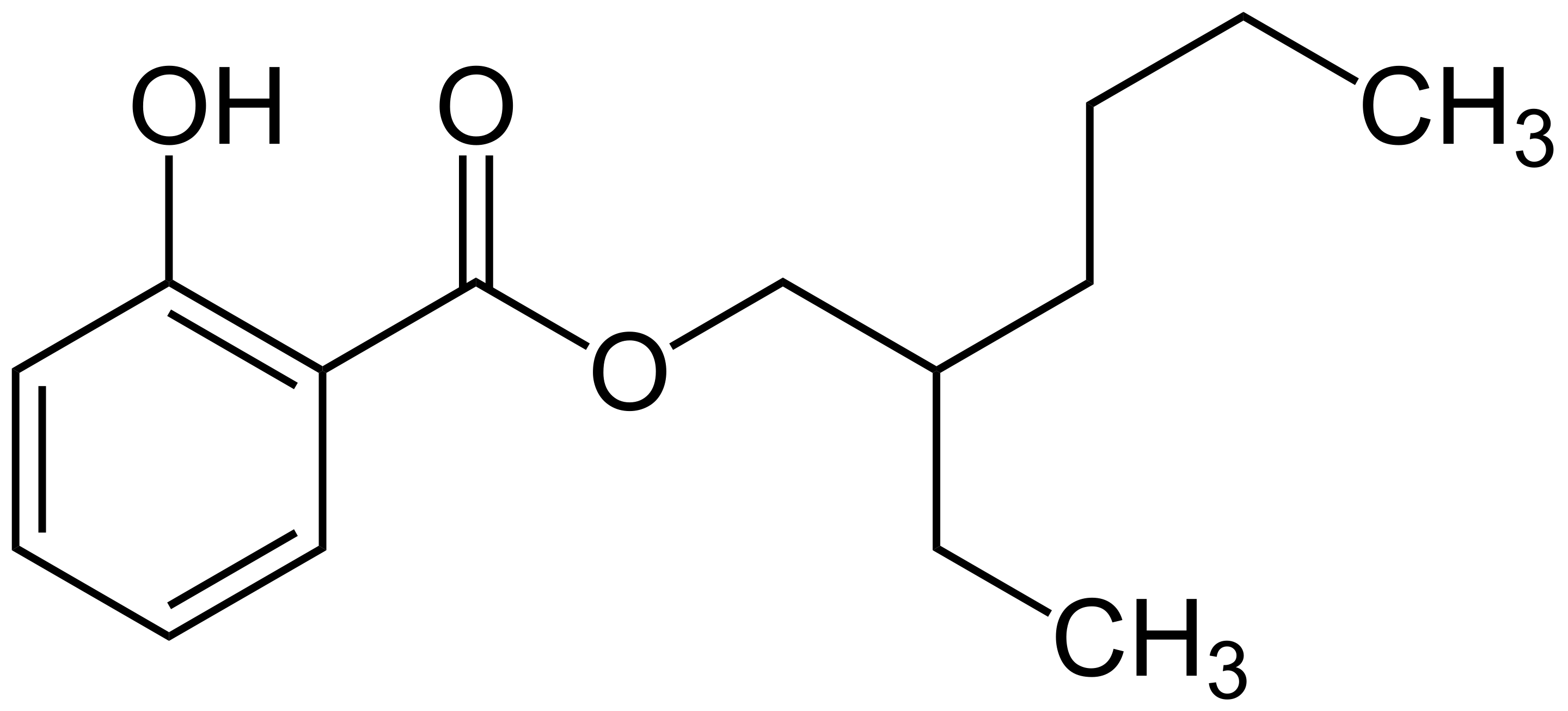 | 118-60-5 | 5.934 | 8.13 |
| Diethylamino hydroxybenzoyl hexyl benzoate  (DHHB) | 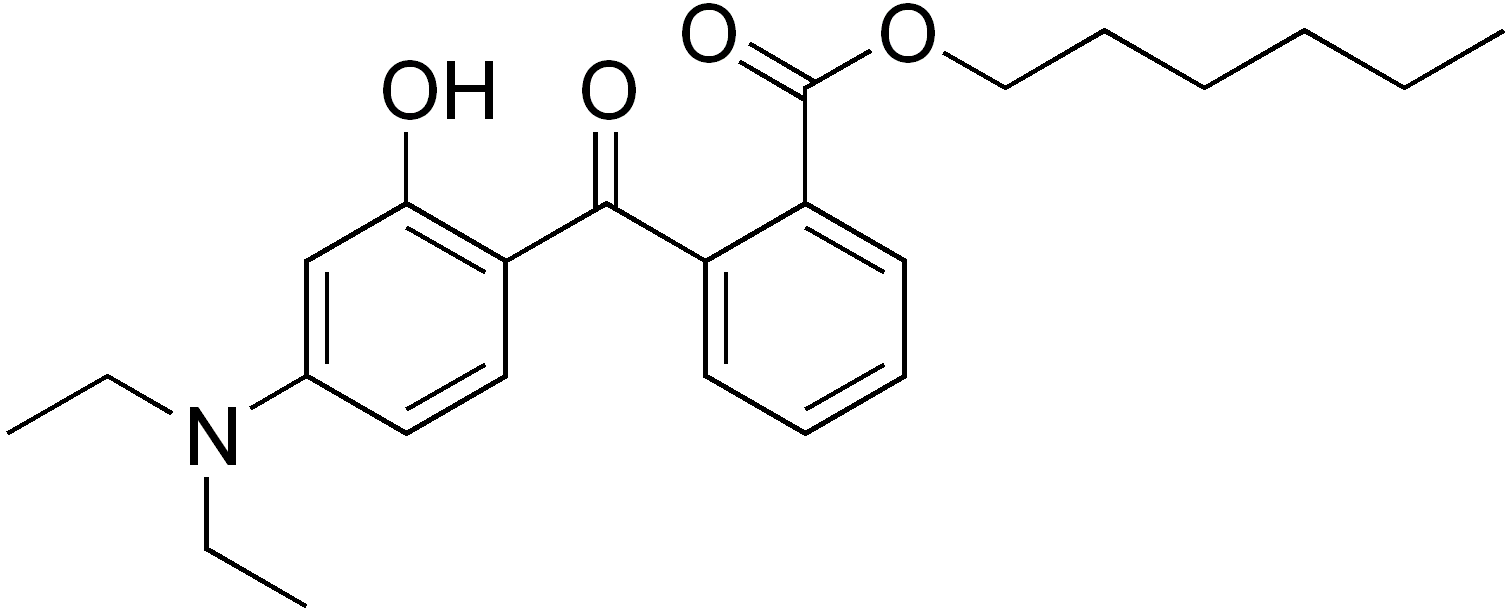 | 302776-68-7 | 6.855 | 7.57 (a) 2.72 (b) |
| Octocrylene  (OC) | 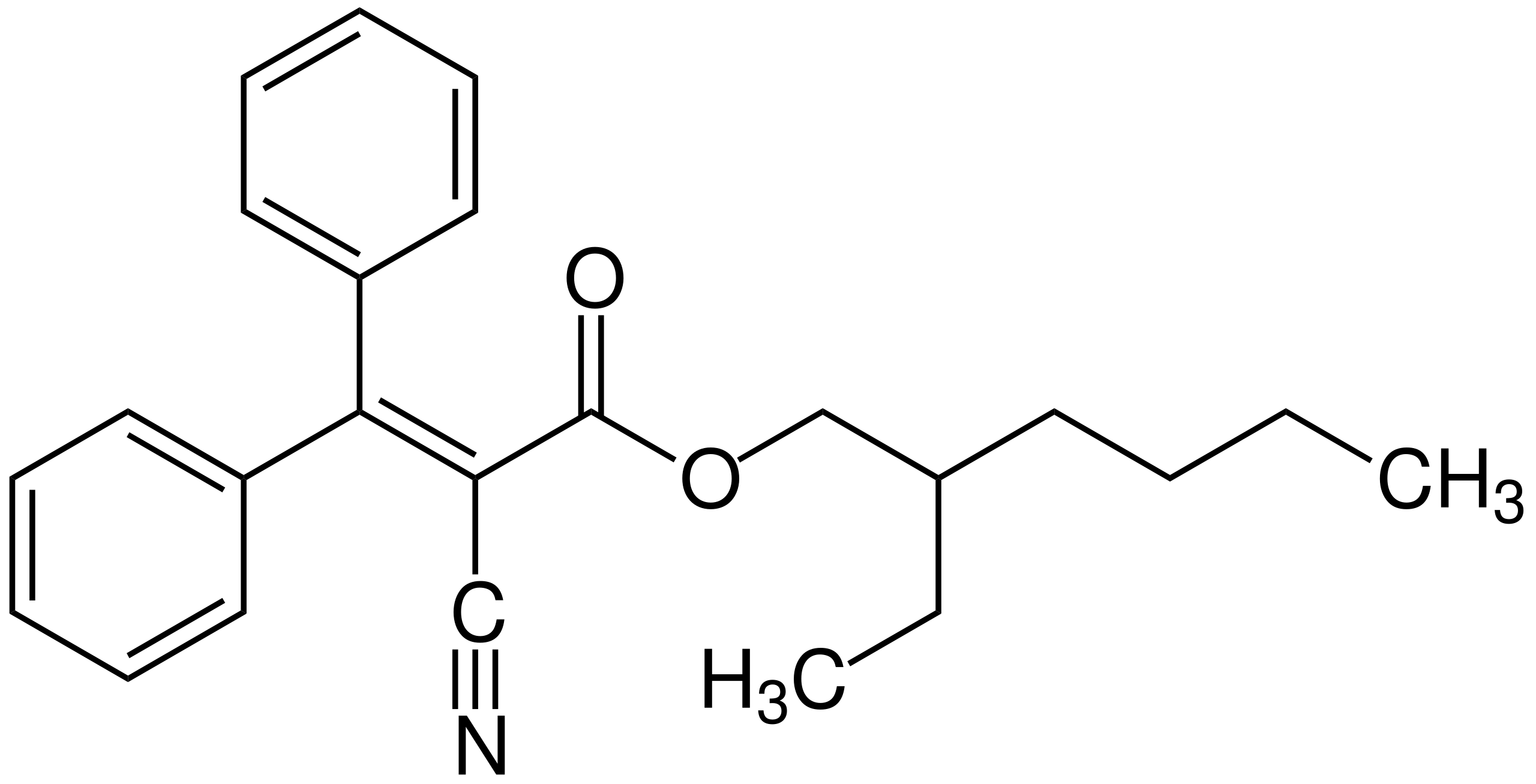 | 6197-30-4 | 6.893 | - |
| Drometrizole trisiloxane  (DTS) | 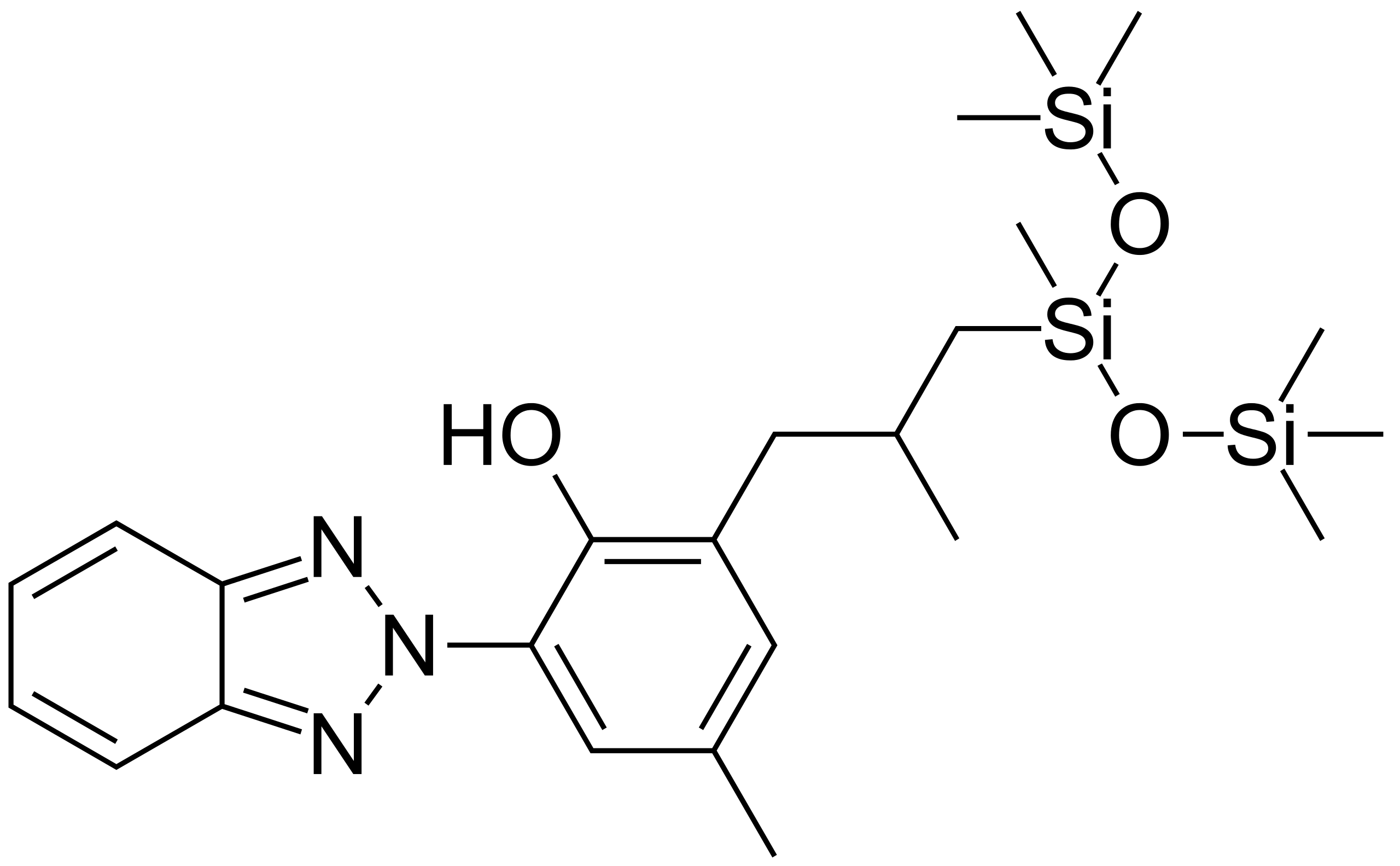 | 155633-54-8 | 8.344 | 8.36 (a) 0.75 (b) |
| Diethylhexyl butamido triazone  (DEBT) | 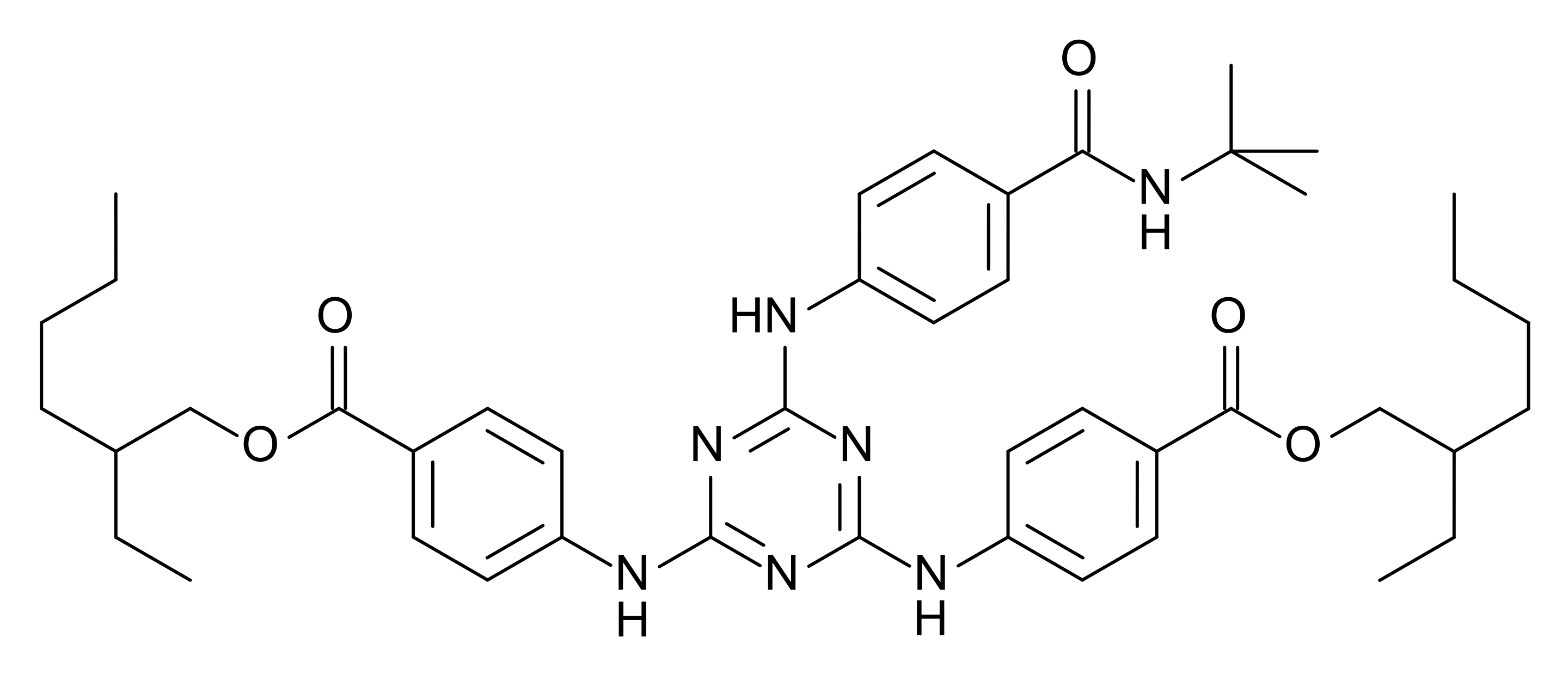 | 154702-15-5 | 12.552 | 15.31 (a) 1.20 (b) |
| Ethylhexyl triazone  (EHT) | 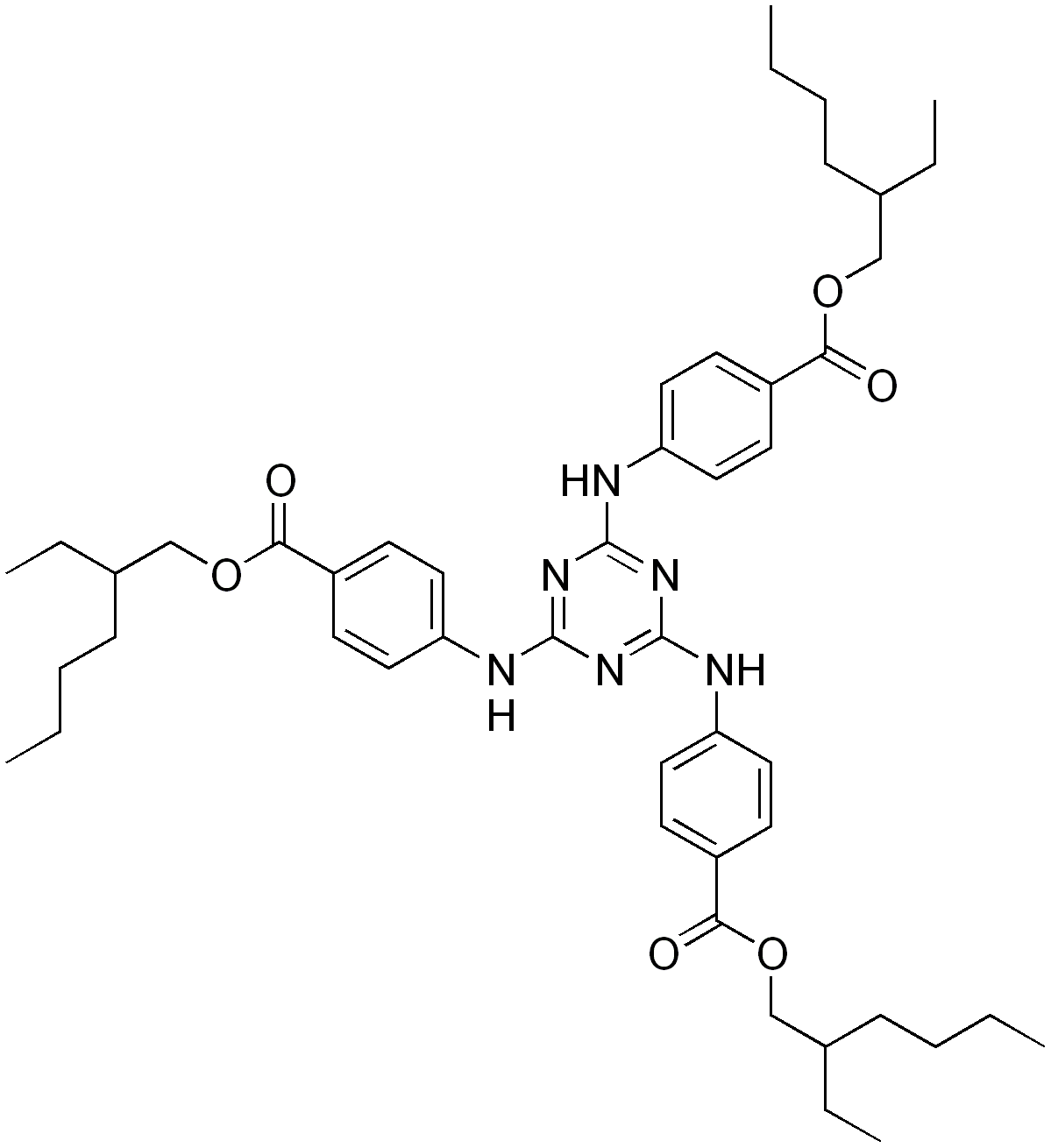 | 88122-99-0 | 16.129 | 1.22 (b) |

^a^ P_ow_: Octanol-water partition coefficient; ^b^ K_a_: Acid-base dissociation constant; (a): acidic group, (b) basic group; Calculated using Advanced Chemistry Development (ACD/Labs) Software V11.02 (© 1994-2020 ACD/Labs)

**Table S2.** Tandem mass spectrometry parameters for the target compounds

| UV filter | Precursor ion (m/z) | Product ion (m/z) ^a^ | Collision energy (V) | Fragmentor (V) |
| --- | --- | --- | --- | --- |
| PBSA | 273.0 | **193**  80 | 26  58 | 150 |
| BZ4 | 307.0 | **211**  80 | 30  46 | 150 |
| BZ3 | 229.0 | **151**  105 | 20  16 | 150 |
| MBC | 255.4 | 212  **171** | 20  16 | 100 |
| BMDM | 311.4 | 161  **135** | 20  20 | 100 |
| IMC | 249.0 | 179  **161** | 4  12 | 100 |
| EHDP | 278.0 | 166  **151** | 20  32 | 150 |
| EHMC | 291.4 | **179**  161 | 2  14 | 100 |
| EHS | 251.3 | **139**  121 | 12  16 | 50 |
| DHHB | 398.5 | **149**  43 | 20  40 | 100 |
| OC | 362.4 | 250  **232** | 2  18 | 150 |
| DTS | 502.8 | **413**  397 | 4  32 | 100 |
| DEBT | 766.0 | 654  **468** | 40  58 | 350 |
| EHT | 824.1 | 487  **308** | 60  86 | 350 |

^a^ The m/z values used as quantifiers are marked in bold
